# Gestational diabetes mellitus prediction? A unique fatty acid profile study

**DOI:** 10.1038/s41387-020-00138-9

**Published:** 2020-09-30

**Authors:** Enitan Ogundipe, Saidee Samuelson, Michael A. Crawford

**Affiliations:** 1 Neonatal Unit, Chelsea and Westminster Hospital & Faculty of Medicine, Imperial College London, 369 Fulham Road, SW10 9NH London, UK; 2 Academic Department of Obstetrics and Gynecology, Imperial College London, Chelsea and Westminster Hospital Campus, 3rd Floor, 369 Fulham Road, SW10 9NH London, UK; 3 Academic Department of Obstetrics and Gynecology (Lipid Biochemistry), Imperial College London, Chelsea and Westminster Hospital Campus, 3rd Floor, 369 Fulham Road, SW10 9NH London, UK

**Keywords:** Fatty acids, Gestational diabetes

## Abstract

**Objective:**

To elucidate whether women at risk of gestational diabetes mellitus (GDM) have a unique fatty acid profile compared to women considered normal healthy controls (NHC).

**Methods:**

Three hundred pregnant women were randomized to a control group (NHC) (*n* = 50) and to one of three high risk groups (*n* = 250), one of which was GDM (*n* = 50). At recruitment participants’ booking bloods were taken and analyzed for lipid profiles. The GDM group’s fatty acid profile is reported here.

**Results:**

GDM women compared to NHC had elevated levels of omega 6 (*n*-6) fatty acids compared to omega 3 (*n*-3) fatty acids (*p* = 0.01), of linoleic acid (LA) to docosahexaenoic acid (DHA) *p* = 0.001, sequentially distorted levels of *n*-6 fatty acids LA and arachidonic acid (ArA) *p* = 0.035, as well as significantly depressed levels of *n*-3 DHA (*p* = 0.01).

**Conclusion:**

This paper shows that GDM women have a unique fatty acid profile with elevated levels of *n*-6 fats, depressed levels of *n*-3 fats and an abnormal pattern of sequential *n*-6 metabolism. This profile probably results from a combination of factors including underexpression and or poor utilization of desaturase enzymes, suboptimal dietary fatty acids intake, poor micronutrient status or dysbiosis of the microbiome. These results help inform development of a clinical predictive tool.

## Introduction

Gestational diabetes mellitus (GDM) is a condition of pregnancy resulting from insulin insensitivity. The presence of GDM is associated with a variety of adverse perinatal outcomes, including but not limited to macrosomia, pre-term birth, pre-eclampsia^1^, and may ultimately influence the onward metabolic health of the resulting child^2^. GDM currently affects around 5% of pregnant women in the UK^3^, however, prevalence is up to 33.5% in South East Asian populations^4^. Presently, the known risk factors to GDM have a predictive accuracy of ~75%^5^, however, future work considering unique predictive markers may improve this.

The primary driver to GDM accounting for greater than 80% of cases is the presence of obesity^6^, where caloric intake exceeds demand, and nutritional status is unbalanced. In the obese model, the continued presence of elevated serum levels of insulin, the elevated existence of pro-inflammatory cytokines/adipokines, chronic inflammation^7^ and reduced circulating adiponectin^7,8^ appear to be related to underlying causes. Polyunsaturated fatty acids (PUFA) have long been considered as anti-inflammatory^7^, and in more recent literature have been directly linked to insulin sensitization and adiponectin upregulation^9,10^, making them an increasingly interesting target for disease prediction and risk reduction.

Fatty acids can be directly consumed through the diet or synthesized sequentially in the liver from their parent fats by a series of elongase and desaturase enzymes. LA is the parent fatty acid to all *n*-6 PUFA, while α-linolenic acid (ALA) is the parent fatty acid to all *n*-3 PUFA, it is from these parent fats that all other *n*-3 and *n*-6 fatty acids are derived. The activity of conversion enzymes is central to the biosynthesis of fatty acids. Conversion enzyme activity can be significantly modified by type of dietary fat intake. Previous works have shown that an increased LA consumption of 15 g/day decreases *n*-3 series ALA to DHA conversion by up to 40%^11^. In addition to dietary fatty acid intake, many other factors including genetics, micronutrient status, and microbiota may also influence fatty acid conversion. Whilst specific enzymatic pathways have been well described, their influence overall on fatty acid conversion, and disease risk is less well understood.

There are many fatty acids, which play a significant role in maternal health and fetal development, however, two fatty acids in particular stand out. One, DHA, an *n*-3 PUFA, is a primary structural lipid within the central nervous system (CNS), mitochondrial membranes and the cerebral cortex^12^, essential for immune function and lipoprotein membrane integrity. The other is ArA, an *n*-6 PUFA, essential for CNS development, placental function, and immune system regulation. In previous preliminary works by Ogundipe’s group^12^, significant differences between fatty acids of NHC and high-risk pregnant women were first described. This paper now explores the significance of these associations, and their relationship with GDM in particular, by comparing the fatty acid profile characteristics of GDM women compared to the NHC group of pregnant women.

Typically a combination of the amount and the ratio of fatty acids within the body are used as a health indicator, where the ArA:DHA ratio can be used as a general marker of inflammation, and is positively correlated with plasma levels of pro-inflammatory adipokines IL-6 and TNF alpha^13^, which are known to play a direct role in insulin insensitivity. Ratios are presently becoming of increasing interest given recent works by Nording et al.^14^ who established the high degree of variability between individuals’ absolute values of red blood cell fatty acids independent of dietary intake alone. The utility of the ArA:DHA ratio has been well explored, however, given the complexity of fatty acid biosynthesis, and the impact of dietary intake much uncertainty remains. Therefore, further exploration is required to elucidate the value of other ratios as markers of disease and to determine unique metabolic differences between varying states of health.

Aims to: (i) establish whether unique fatty acid ratios in the GDM state exist

(ii) determine the utility of fatty acid profiling as an early predictor of GDM.

The data that form the basis of this paper are from the FOSS study^12,15^, the focus of which was to determine the influence of fish oil supplementation on pregnancy outcomes, of which GDM was an outcome group. This paper reports on the prediction of GDM as a pregnancy outcome when compared to the pregnant NHC women.

## Methodology, materials and methods

**Fig. 1:**
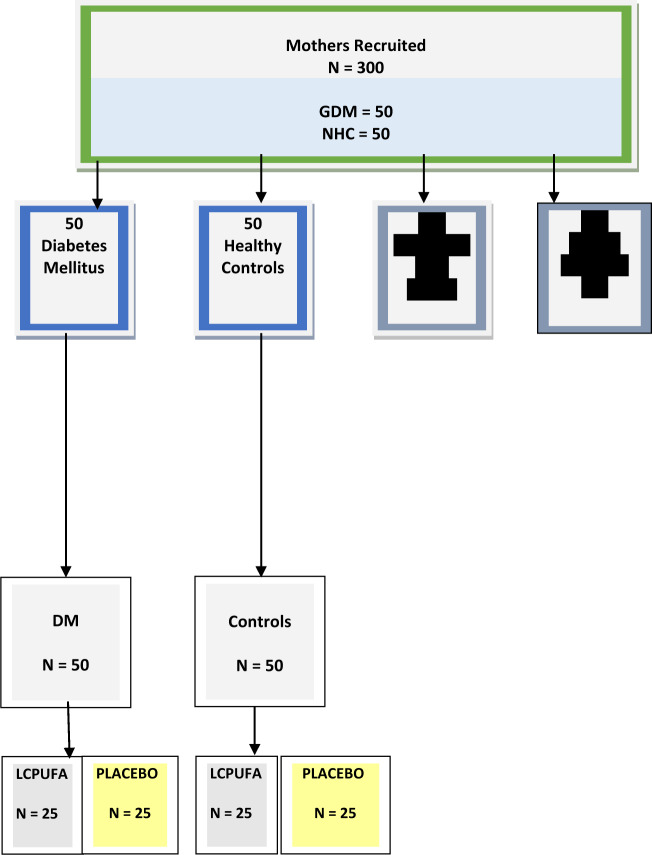
Participant Recruitment and Supplementation Sub-Group: GDM vs NHC.

A double blind randomized placebo controlled trial, performed with participants recruited from Chelsea and Westminster Hospital London antenatal clinic, at booking. Three hundred women took part in the FOSS study^12^ and were randomized to a control group (NHC) (*n* = 50) and to one of the three other high-risk groups (*n* = 250) that included GDM (*n* = 50) see Fig. 1. For the purposes of this report we are exclusively commenting on GDM women (*n* = 50). Women at high risk of GDM were selected to form part of this sub group based on the following inclusion criteria: older than 35, South East Asian or North African descent, previous GDM, family history of Type II DM, raised body mass index (BMI) > 30 and previous macrosomic infant. *At recruitment* participants’ bloods were taken prior to supplementation, and analyzed for lipid profiles. Erythrocyte total lipids were extracted by the Folch method^16^ and fatty acid compositions were analyzed by capillary gas chromatography. The results stated refer to women as selected by our inclusion criteria. GDM was ultimately determined in accordance with standard diagnosis criteria as previously described by NICE^3^.

Written consent was obtained from all participants. Ethical approval for the study was given by the East London Research Ethics Committee (07/H0704/99).

Trial registration No: ISRCTN 24068733.

## Statistical analysis techniques

Student *t* test was used to compare continuous data. The differences were compared using the unpaired two-tailed student *t* test. Normality of the data was checked graphically with frequency distribution numerically and also utilizing histogram plots. Chi-squared test was used for categorical variables within the demographic information, SPSS for Windows (Release no 22) [IBM SPSS Statistics V22.0 (SPSS, IBM, Chicago, IL, USA)] was used to perform statistical analysis. Statistical significance was taken to be *p* value <0.05.

## Results

### Demography of participants

#### Table 1a, b: Anthropometric and demographic characteristics of participants

**Table 1:** (a) Maternal characteristics: comparison of GDM versus NHC. (b) Sociodemography by maternal sub group: GDM versus NHC.

(a)			
Characteristics	GDM *N* = 48	NHC *N* = 48	
	Mean (±SD)	Mean (±SD)	*p* Value
Maternal age	32.40 (6.51)	33.31(5.14)	0.449
Maternal height (cm)	162.54 (7.75)	165.84 (6.53)	0.026
Maternal weight (kg)	78.71 (17.78)	68.40 (11.07)	0.001
Maternal BMI	29.73 (5.52)	25.0 (3.91)	0.000

(b)			
	GDM *n* = 49	NHC *n* = 51	*p* Value
Maternal ethnicity			0.049
White	20	35	
Black	12	7	
Asian	7	4	
Mixed/other	10	5	
Marital status			0.008
Married	33	30	
Co-habiting	6	19	
Single	9	3	
Divorced	0	0	
Maternal educational level			0.010
Primary only	7	2	
Secondary	24	14	
College/University	14	27	
Postgraduate	4	9	
Maternal occupation group			0.004
Professional	12	26	
Non professional	12	16	
Unemployed	25	11	
Paternal ethnicity			0.257
White	22	31	
Black	12	6	
Asian	6	4	
Mixed/other	9	11	
Paternal educational level			0.258
Primary only	3	0	
Secondary	31	34	
College/University	10	11	
Postgraduate	3	6	
Paternal occupation group			0.081
Professional	19	29	
Non professional	21	20	
Unemployed	9	3	
Smoking in pregnancy			0.457
No	47	49	
Yes	2	4	
Alcohol in pregnancy			0.605
No	48	51	
Yes	1	2	

Chi-squared (Table b) or Student *t* test (Table a) utilized.

Women in the GDM group were significantly heavier and shorter with higher BMI than the normal healthy control (NHC) group. There were no statistically significant differences in age at time of study (Table 1a).

There were statistically significantly more GDM women of lower socioeconomic status compared to the control group, with lower educational achievement and a significantly higher percentage of unemployment in the GDM group. No difference in social habits of smoking or alcohol use was shown. By contrast, paternal sociodemographic status of either group did not differ (Table 1b).

### Fatty acid profiles—Table 2: fatty acid profiles of GDM and NHC women

**Table 2:** Fatty acid profile by maternal sub group: GDM versus NHC.

Fatty acid	GDM *n* = 47	NHC *n* = 46	
	Mean (±SD)	Mean ± SD	*p*
Palmitic acid (PA)	24.61 (1.38)	23.89 (1.39)	0.014
Linoleic acid (LA)	14.22 (2.20)	13.31 (1.67)	0.028
Eicosadienoic acid (EDA)	0.03 (0.02)	0.04 (0.02)	0.017
Adrenic acid (AdA)	2.10 (0.40)	1.91 (0.38)	0.021
Docosapentaenoic acid (DPA)	1.59 (0.37)	1.81 (0.32)	0.003
Docosahexaenoic acid (DHA)	4.35 (1.14)	4.92 (0.91)	0.010
Arachidonic acid (ArA)/ DPA	16.75 (2.27)	17.70 (1.89)	0.031
LA/ArA	1.18 (0.32)	1.06 (0.21)	0.035
LA/DHA	3.55 (1.30)	2.80 (0.65)	0.001
ArA/DHA	3.01 (0.72)	2.68 (0.51)	0.012
DHA/EPA	8.92 (4.23)	7.25 (2.45)	0.022
*n*-6/*n*-3	4.69 (1.23)	3.92 (0.73)	0.000
LC *n*-6/LC *n*-3	2.66 (0.65)	2.30 (0.45)	0.003
MUFA/*n*-3	2.88 (0.93)	2.44 (0.66)	0.011
MUFA/LC n-3	3.07 (1.03)	2.58 (0.72)	0.100

Results of fatty acids shown were given as a mean percentage of total fatty acids in erythrocytes. When compared to NHCs, GDM women are shown to have elevated saturated fat (palmitic acid), *n*-6 PUFAs including; linoleic acid (LA), adrenic acid (AdA) and ratios of LA:ArA, *n*-6 to *n*-3, LA:DHA and ArA:DHA and LC*n*-6:LC*n*-3. In addition, GDM women had lower levels of the *n*-3 PUFAs, docosapentaenoic acid (DPA), ArA, DHA, n-3 MUFA when compared to the NHCs (Table 2).

## Discussion

This unique study describes the patterns and ratios between fatty acids as seen in early pregnancy in women at risk of GDM when compared to NHC. We have chosen to discuss ratios in addition to absolute values, given the high degree of variability of intake and metabolic rates in individuals. Ratios give a clearer understanding of specific metabolic insufficiencies, which may underpin the GDM state. Previous works have suggested hepatic enzyme conversion of fatty acids can vary between subjects by up to 70%^17^, however, there has been no singular primary enzymatic insufficiency described within GDM. This work that describes fatty acid ratios uniquely associated with GDM, may now be incorporated into a new predictive tool, that may also increase intervention potential. Presently, there is a low patient value to existing early interventions, however, no such intervention has specifically considered targeted fatty acid supplementation of fatty acid profile modification, this study could help to inform the basis of such onward interventions.

### Sociodemographic data

Previously established and well described risk factors to GDM including maternal BMI and maternal ethnicity maintained relevance, as the strongest predictor to the disease state, where BMI was the most significant marker. Interestingly maternal age was not significant within our cohort, however, maternal education and occupation emerged as factors that were relevant to the onset of GDM. This is something that was previously reported by Bertolotto et al.^18^ who described an association between maternal educational level and BMI but not directly with GDM onset. Our study showed that paternal ethnicity, education, and occupation are not significantly different between the groups hence alluding to the fact that the GDM risk is significantly linked to maternal status.

### Fatty acid markers of GDM

GDM women had elevated ratios of *n*-6/*n*-3 PUFA, when compared to the NHC group. This is consistent with previous observational studies which have noted that the diet of obese individuals is typically deficient in *n*-3 fats and rich in *n*-6 fats^19^. Lower dietary intake of parent *n*-3 fatty acid ALA, coupled with direct low dietary consumption of sequentially synthesized fatty acids may be a causative factor in the significantly lower levels by ratio of *n*-3 fatty acids. Low maternal levels of parent *n*-3 fatty acids and those that are sequentially synthesized including DHA may be directly significant to the pathogenesis of GDM. Low DHA may compromise the body’s ability to regulate adiponectin and synthesize DHA-derived anti-inflammatory agents, maresins, protectins and resolvins^20^. Working synergistically these agents act to inhibit TNF-alpha secretion and upregulate adiponectin production. Further exploration of this is beyond the scope of this study but will be considered in our future work.

GDM women had significantly elevated amounts of parent *n*-6 fatty acid LA, however, uniquely to GDM, many of LA sequential fatty acid metabolites were not elevated. In normal fatty acid synthesis *n*-6 parent fat LA converts to ArA in a series of elongase and desaturase reactions. In GDM women, an elevated ratio of LA/ArA highlights an inconsistency in the metabolic pathway and suggests metabolic insufficiencies in desaturase or elongase conversion enzymes, unique to the GDM profile.

ArA converts via elongase to AdA, which converts to DPA via desaturase. The nature of this metabolic pathway predicts that elevated precursors would lead to elevated metabolites, this however, is not the case in the GDM state. When considering the ratios of these fatty acids, we see a very unusual pattern. ArA/AdA was depressed meaning higher absolute values of a sequential metabolite existed when compared to its parent fat. This arguably could be caused by direct dietary consumption, however, onwardly there exists an elevated ratio of AdA/DPA (its sequential metabolite), which only requires the presence of desaturase enzymes to convert. This undulating trough-to-peak-to-trough pattern suggests a functional deficit in the GDM state with hepatic desaturase enzyme function.

The link between insulin sensitivity and modification of fatty acid metabolism through augmented desaturase enzyme function has previously been explored by Arbo et al.^21^ however, with GDM this would suggest the women had pre-existing diabetes or insulin insensitivity prior to gestation in order for such a profile of fatty acids to exist in early pregnancy.

Uniquely our data show that GDM women at booking prior to any supplementation have a significantly reduced proportion of DPA, the precursor to DHA, when compared to NHC women. In normal fatty acid metabolism, *n*-6 fatty acid AdA, which was elevated in the GDM group, is converted to DPA via the Δ^4^ desaturase enzyme, and is ultimately converted to DHA by another desaturation step. Logically, elevated amounts of precursor fatty acids should lead to elevated amounts of their products, however, in women with GDM, a similar pattern of peaks and troughs exists once again, suggesting a malfunction in the chain of fatty acid synthesis and conversion again with respect to denaturization.

The expression and activity of the Δ^6^- and Δ^5^-desaturase enzymes responsible for *n*-3 and *n*-6 fatty acids conversion, is up-regulated in pregnancy to increase the rate of hepatic ArA synthesis^22^, required to meet maternal demand. By contrast Δ^6^- and Δ^5^-desaturase enzyme production and activity is depressed in diabetes due to insulin insufficiency or resistance^23^. Therefore, the conversion of *n*-6 precursors to ArA in diabetic pregnant women may be uniquely compromised and affect ArA status. This hypothesis may account for the prevalence of elevated *n*-6 precursors, but reduced ArA:DHA ratio in at-risk GDM women.

Previous studies have shown the factors that regulate the synthesis of *n*-3 and *n*-6 fatty acids from parent fatty acids LA and ALA are affected by pre-existing metabolic dysregulation and elevated saturated fat levels as seen in our GDM group. It has been shown^24^ that insulin sensitivity and adiposity are independent factors that influence activity of enzymes responsible for the regulation of essential fatty acid (EFA) metabolism. In disease states characterized by metabolic dysregulation, including insulin resistance, diabetes and obesity, impaired Δ^5^-desaturase, Δ^6^-desaturase, and elongase activities are previously described however never fully characterized in the GDM model. Our data go some way to elucidating a fuller overview of the GDM fatty acid profile.

To summarize, women at risk of GDM have a unique and interesting profile of fatty acids undulating throughout the sequential chain. As described in detail above it is possible to attribute this profile to an insufficiency or underexpression of desaturase enzymes. Desaturase enzyme function is known to be altered by pregnancy, existing insulin resistance, saturated fat intake, and overall dietary fat intake as well as other potential factors including gut microbiota and micronutrients. Zinc is known to play a major role in a variety of biological functions including immunity, protein synthesis, enzyme function, and metabolism/lipid metabolism^24^. However, the dietary absorption of zinc is influenced by several factors including the presence of EFA^25^, following their conversion to prostaglandins, and the competitive inhibition of zinc uptake by dietary iron^26^. Zinc is synergistically associated with fatty acids, as it facilitates the conversion of LA to GLA, the mobilization of DihommogammaLA (DGLA) and the conversion of DGLA to ArA and ArA mobilization^27^. Therefore, low levels of EFA in the pre-pregnancy diet will limit zinc absorption, resulting in zinc deficiency that will lead to the poor conversion of fatty acids LA to ArA and to an elevated LA/ArA ratio as observed in the GDM group^25–27^. A full discussion on the micronutrient profile of women at risk of GDM is beyond the scope of this paper.

### Conclusions

This study provides evidence to support the hypothesis that fatty acid profiles of pregnant women, at risk of GDM, at antenatal booking, are unique and significantly different to those of healthy pregnant women. GDM women were shown to have significantly elevated levels of *n*-6 fatty acids, depressed levels of *n*-3 fatty acids and an abnormal pattern of sequential *n*-6 metabolism. The findings presented here provide unique evidence that may be used to inform the production of a clinically applicable tool, able to predict GDM risk in early pregnancy.
